# A *Drosophila* ABC Transporter Regulates Lifespan

**DOI:** 10.1371/journal.pgen.1004844

**Published:** 2014-12-04

**Authors:** He Huang, Ying Lu-Bo, Gabriel G. Haddad

**Affiliations:** 1 Department of Pediatrics (Division of Respiratory Medicine), University of California San Diego, La Jolla, California, United States of America; 2 Rady Children's Hospital San Diego, San Diego, California, United States of America; Stanford University School of Medicine, United States of America

## Abstract

MRP4 (multidrug resistance-associated protein 4) is a member of the MRP/ABCC subfamily of ATP-binding cassette (ABC) transporters that are essential for many cellular processes requiring the transport of substrates across cell membranes. Although MRP4 has been implicated as a detoxification protein by transport of structurally diverse endogenous and xenobiotic compounds, including antivirus and anticancer drugs, that usually induce oxidative stress in cells, its *in vivo* biological function remains unknown. In this study, we investigate the biological functions of a *Drosophila* homolog of human *MRP4*, *dMRP4*. We show that *dMRP4* expression is elevated in response to oxidative stress (paraquat, hydrogen peroxide and hyperoxia) in *Drosophila*. Flies lacking *dMRP4* have a shortened lifespan under both oxidative and normal conditions. Overexpression of *dMRP4*, on the other hand, is sufficient to increase oxidative stress resistance and extend lifespan. By genetic manipulations, we demonstrate that *dMRP4* is required for JNK (c-Jun NH_2_-terminal kinase) activation during paraquat challenge and for basal transcription of some JNK target genes under normal condition. We show that impaired JNK signaling is an important cause for major defects associated with *dMRP4* mutations, suggesting that *dMRP4* regulates lifespan by modulating the expression of a set of genes related to both oxidative resistance and aging, at least in part, through JNK signaling.

## Author Summary

The drug transporters are often known for their ability to transport different physiological-related compounds across cell membranes. Although the abnormal up-regulation of some these transporters is believed to be the common cause of the clinic problem called drug resistance, the biological functions of these transporters remain largely unknown. Here we show that a *Drosophila* homolog of the mammalian drug transporter plays a role in lifespan regulation. Mutations of this gene increase the sensitivity to oxidative stress and reduce lifespan, while overexpression of this gene increases resistance to oxidative stress and extends lifespan. By molecular and genetic analyses, we have linked functions of this gene to a key signaling transduction pathway that has been known to be important in lifespan regulation.

## Introduction

In *Drosophila*, one important feature of the aging process appears to be the similarity between the changes in gene expression that occur during aging and oxidative stress response [1], [2], [3]. For instance, the up-regulation of genes encoding for some chaperones and/or detoxification agents in response to oxidative stress has been found to highly correlate with the aging process [1], [2], [3]. Hsp proteins may promote longevity by facilitating the clearance of damaged proteins that accumulate during aging [4]. Another example is the JNK signaling pathway which can be triggered by a variety of insults, including oxidative stress, and has been shown to be a genetic determinant of aging in *Drosophila* [5]. Mutations in the JNK cascade increase stress sensitivity and lead to shortened lifespan. Conversely, flies with increased JNK activity can sustain oxidative stress and live longer [6]. Although genome-wide surveys [1] are powerful and have linked a set of genes between stress response and aging, the majority of them have not been tested experimentally for lifespan; some genes involved in both processes may still be missing by genome-wide surveys. Here we report that a new gene, namely *dMRP4*, which has not been reported on the survey list [1], clearly plays a role in both aging process and oxidative stress.

The multidrug resistance-associated protein 4 (MRP4) belongs to the subfamily C (also known as ABCC) of the ATP-binding cassette (ABC) transporter protein family. It has been classified as a detoxification protein that is implicated in transport of structurally diverse endogenous and xenobiotic compounds, including antivirus and anticancer drugs that usually induce oxidative stress in cells and lead to toxicity [7], [8], [9]. *MRP4* mRNA and protein are widely expressed in many tissues of mammals including humans [10], suggesting that this transporter may be involved in different physiological processes. However, several recent studies have shown that mammalian *MRP4* is not essential for development, since *MRP4*-knockout mice are viable and do not reveal any abnormalities [11], [12], [13], [14]. Therefore, the biological function of *MRP4* remains largely unknown.

MRP-associated drug resistance has represented an important clinical problem in the treatment of cancers. Some cancer cells seem to adopt a survival strategy to protect against chemotherapy-induced oxidative stress by increasing transport of chemotherapeutics out of cells, as a result of induction of MRP, including MRP4 [15], [16], [17], [18], [19]. Indeed, up-regulation of MRP4 expression has been linked to a variety of human cancers [20], [21], [22], [23], [24]. The induction of hepatic MRP4 by oxidative stress has also been observed in mammalian liver injury after chemical treatments and this response appears to be regulated primarily at a transcriptional level [25], [26]. However, oxidative stress-inducing agents do not always induce MRP4 [27], [28], [29], [30], raising the possibility that the induction of MRP4 expression during oxidative stress may be agent-dependent and/or cell type-specific. Furthermore, no study has attempted to address whether MRP4 is required for general oxidative stress resistance at a whole organismal level.

We have previously identified the *Drosophila* homolog of mammalian *MRP4*, called *dMRP4*, during an unbiased screen for genes whose overexpression causes an abnormal response to hypoxia in adult flies [31]. *dMRP4* encodes a protein sharing 43% overall amino acid identity and 63% similarity with the human MRP4 [32], [33]. In this study, we have investigated the possible involvement of *dMRP4* in resistance to oxidative stress. By genetic manipulation, we present evidence that *dMRP4* is associated with changes in lifespan under both oxidative stress and normal conditions, likely through a mechanism that is linked to JNK signaling in *Drosophila*.

## Results

### *dMRP4* is an oxidative stress-responsive gene and is required for oxidative stress resistance

**Figure 1:**
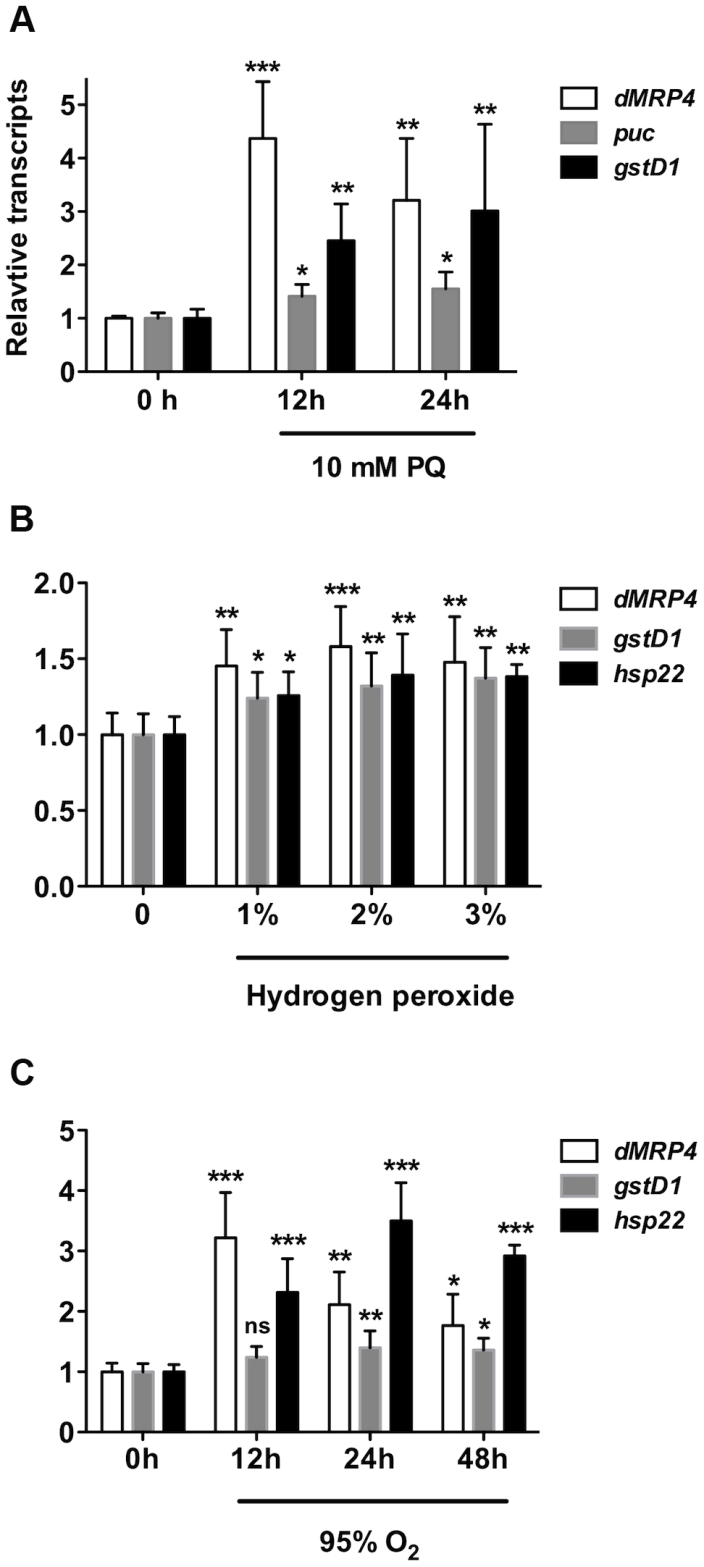
*dMRP4* is up-regulated in response to oxidative stress. (A) Quantitative RT-PCR analyses of RNA isolated from wild-type flies (*w^1118^*) after exposed to paraquat (A), hydrogen peroxide (B,) or hyperoxia (C) for indicated times. Data is showed as means ± S.D. from at least 5 independent experiments. One way ANOVA followed by post hoc *t*-test: * *p*<0.05, ** *p*<0.01, *** *p*<0.001, ns: No significance (*p*>0.05).

To test our hypothesis that the expression of *dMRP4* may be regulated by oxidative stress in *Drosophila*, we first analyzed *dMRP4* transcriptional activity in response to oxidative stimuli by feeding flies with paraquat, which generates superoxide in mitochondria [34] and has been widely used as an oxidative stress inducer *in vivo*. The expression of *dMRP4* was strongly induced in wild-type flies fed with 10 mM paraquat for 12 hours (Fig. 1A). Similar induction patterns were observed in parallel with two known oxidative stress-responsive genes [3], [6], [35], *puc* (*puckered*) and *gstD1* (*glutathione s transferase D1*). To test whether *dMRP4* responds to other oxidative stressors, we analyzed its transcriptional changes in flies treated with hydrogen peroxide as well as hyperoxia. Up-regualtion of *dMRP4* was clearly observed after hydrogen peroxide or hyperoxia treatment, in parallel with two known up-regulated markers, *gstD1* and *hsp22*, under these conditions [1] (Fig. 1B–C). These results indicate that *Drosophila dMRP4* is a *bona fide* oxidative stress-responsive gene.

**Figure 2:**
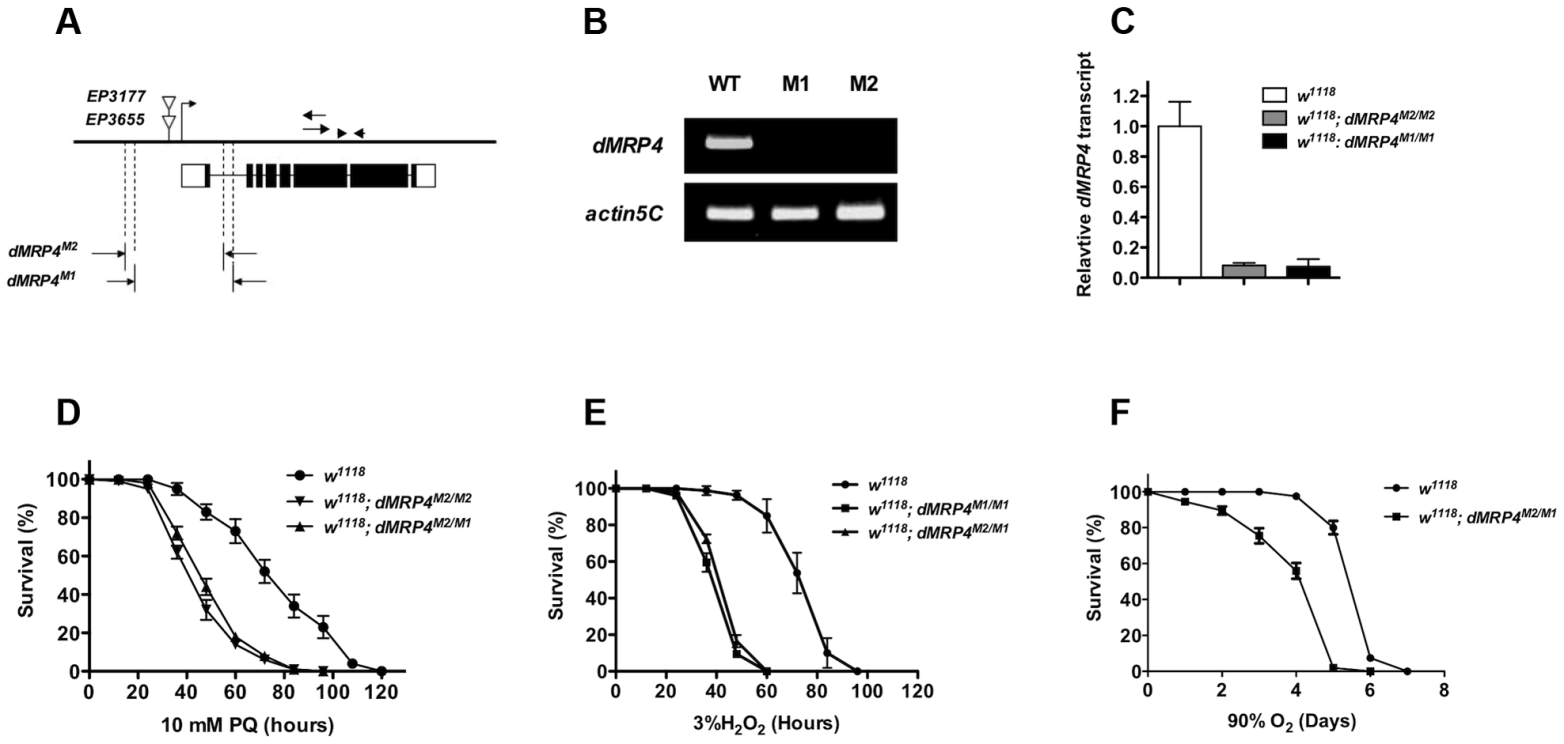
*dMRP4* is required for oxidative stress resistance. (A) Molecular analysis of *dMRP4* mutants. The solid bar represents the genomic region of *dMRP4*. The bent arrow indicates the transcription start site of *dMRP4* gene. The open triangles show the insertion positions of *EP3655* and *EP3177*. Open boxes below the solid bar represent exons of *dMRP4* transcript and filled boxes indicate the encoding protein sequences. The span of both deletions was determined by sequencing the corresponding regions with specific primers. The arrows were primers for *dMRP4*-related semi-quantitative RT-PCR and arrowheads for qt-PCR experiments. The deleted sequences were described in **Materials and Methods**. (B) Expression of *dMRP4* mRNA in two mutant alleles. Semi-quantitative RT-PCR was used to determine the levels of *dMRP4* mRNA expression. *dMRP4* mRNA was under-detectable in *dMRP^M1/M1^* (M1) or *dMRP^M2/M2^* (M2). *Actin5C* served as an internal standard. (C) qt-PCR analysis of *dMRP4* mRNA in two *dMRP4* alleles. (D) Effects of paraquat-induced oxidative stress on *dMRP4* mutant flies (n = 180 for each group). (E) Effects of hydrogen peroxide-induced oxidative stress on *dMRP4* mutant flies (n = 200 for each group). (F) Effects of hyperoxia-induced oxidative stress on *dMRP4* mutant flies (n = 200 for each group). Error bars represent S.E.

To test whether *dMRP4* indeed might play a role in oxidative stress resistance, we generated two mutations by excision of two independent EP elements near the *dMRP4* gene (Fig. 2A). Analysis of the *dMRP4* expression by RT-PCR indicated that *dMRP4* RNA was undetectable in these mutants (Fig. 2B). However, the more sensitive assay with qt-PCR revealed about 8% *dMRP4* mRNA retaining in both homozygous mutations (Fig. 2C). Currently it is not clear if this transcript residual was resulted from splice forms of the predicted full length mRNA or from an alternative transcription start site of the remaining *dMRP4* transcript after the truncation. Nevertheless, these results indicate that the two *dMRP4* alleles represent strong loss-of-function mutations. In addition, flies homozygous for both mutations were viable and fertile, suggesting that *dMRP4* may not be an essential gene for development. However, it cannot be ruled out that the remaining residual in these mutations might still retain some vital function during development.

To address whether induction of *dMRP4* is required for defense against oxidative stress, we monitored the survival of adult flies treated with three most commonly used oxidative stressors: paraquat, hydrogen peroxide, or hyperoxia. In each condition the two *dMRP4* alleles or their transheterozygous combination displayed similar and reproducible phenotypes: flies lacking *dMRP4* reduced profoundly their viability under oxidative stress relative to controls (Fig. 2D–F, Log-rank test, *p*<0.001). These results demonstrate that wild-type *dMRP4* is required for oxidative stress resistance in *Drosophila*.

### *dMRP4* is required for JNK-dependent induction of gene expression

**Figure 3:**
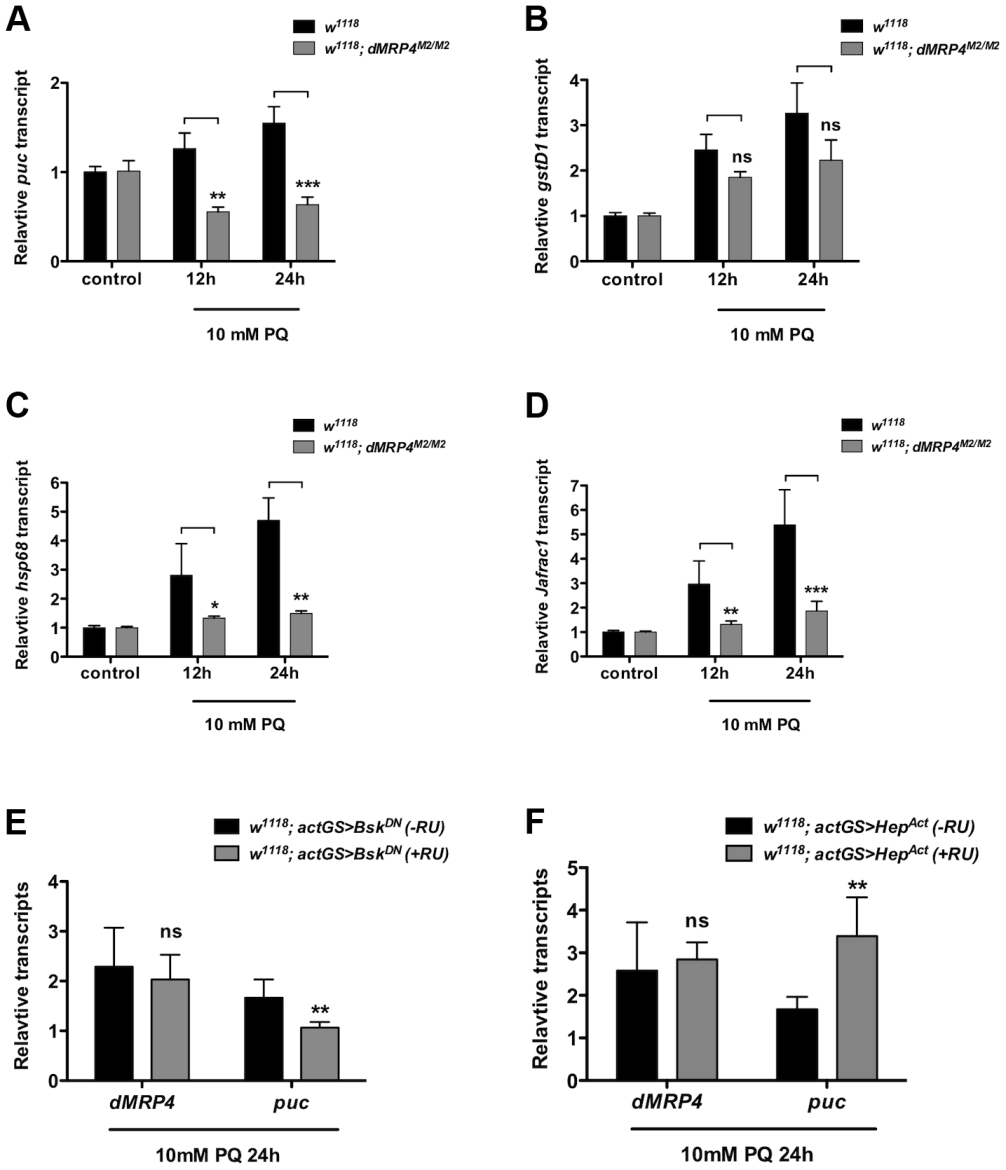
*dMRP4* is required for JNK-mediated gene expression under oxidative stress. qt-PCR analysis of mRNA isolated from *WT* flies (*w^1118^*) after exposed to 10 mM paraquat (PQ) for indicated time points. (A–D) Relative mRNA levels were compared between *w^1118^* and *dMRP4* to the respective controls (no paraquat feeding) where the basal values were set at 1.0. (E) Relative mRNA levels of *dMRP4* from flies carrying *actGS-Bsk^DN^* were compared between RU486 feeding (+RU, 150 ug/ml) and non-feeding groups [75]. (F) Relative mRNA levels of *dMRP4* from flies carrying *actGS-Hep^Act^* were compared between RU486 feeding (+RU, 150 ug/ml) and non-feeding groups [75]. *puc* expression served as a marker for JNK activity in response to paraquat. Data was presented as means ± SD from 3–5 independent experiments. Student's *t*-test: * *p*<0.05, ** *p*<0.01, *** *p*<0.001. ns: No significance (*p*>0.05).

Oxidative stress is known to activate a protective program involving induction of a number of stress-responsive genes in cells [3], [6], [35], [36]. JNK signaling is activated in response to oxidative stress and is a major genetic factor in control of oxidative stress tolerance and aging process [3], [6], [35], [36], [37]. Since *puc* (a phosphatase inhibitor of JNK) is often used as a marker for activation of the JNK pathway [3], [6], [35], [36], [38], we tested whether there were any differential expression changes of JNK signaling by examining *puc* induction in *dMRP4* mutant flies fed with paraquat. Compared to the pattern in wild-type flies, *puc* expression was completely diminished in *dMRP4* mutant flies under oxidative stress (Fig. 3A). To further evaluate whether *dMRP4* might play a general role in JNK signaling, induction of other JNK-mediated marker genes, such as *gstD1* [6], *hsp68* and *Jafrac1*, was also examined. Although expression of all these marker genes was induced in wild-type flies after paraquat feeding, their induction, with exception for *gstD1*, was significantly reduced in the *dMRP4* mutant flies (Fig. 3C–D), indicating that activation of JNK signaling by oxidative stress requires a wild-type *dMRP4* function. Because flies deficient for JNK signaling become more susceptible to stress [6], a phenotype resembling what we have observed with flies deficient for *dMRP4*, impairment of JNK signaling in *dMRP4* mutants may be an important cause for increased lethality when animals face oxidative insults. There was also a possibility that *dMRP4* itself may be a component of the JNK pathway.

To test whether *dMRP4* might be a component of the JNK pathway, we examined *dMRP4* response in flies with reduced activities of JNK signaling by the expression of a dominant negative form of Bsk (*Bsk^DN^*) (Basket, a *Drosophila* homolog of JNK). *Bsk^DN^* can mimic *bsk* mutant phenotypes in flies and cells [39]. In this experiment, *Bsk^DN^* expression was induced in adult flies by *actin-GeneSwitch-Gal4 (actGS-Gal4)*, a RU486-mediated system [40] that drives ubiquitous expression in whole fly. In the presence of drug RU486, *Bsk^DN^* expression was activated from the UAS driven transgene. The relative mRNA levels from RU486-fed flies were compared to control flies carrying the same induction system (*actGS>dMRP4*) without drug feeding. Inhibition of JNK activity by *Bsk^DN^*, as shown by *puc* expression, did not repress *dMRP4* induction in response to paraquat (Fig. 3E), indicating that JNK signaling is not required for *dMRP4* induction under this stress. Next we asked whether stimulation of JNK signaling might influence *dMRP4* induction. This was achieved by conditionally expressing an activated version of Hep (*Hep^Act^*) (*hemipterus*, a *Drosophila* homolog of JNKK). *Hep^Act^* has been shown to be a JNK gain-of-function mutant [39]. Constitutive activation of JNK signaling by *Hep^Act^* did not change *dMRP4* expression in paraquat-fed flies relative to controls (Fig. 3F). These results indicate that unlike those direct targets of JNK, *dMRP4* induction by paraquat is independent of JNK activity, and therefore *dMRP4* is not a direct component, but instead acts in parallel on a signaling that perhaps only regulates expression of some downstream effectors, of the JNK pathway.

### Overexpression of *dMRP4* in adults confers oxidative resistance

**Figure 4:**
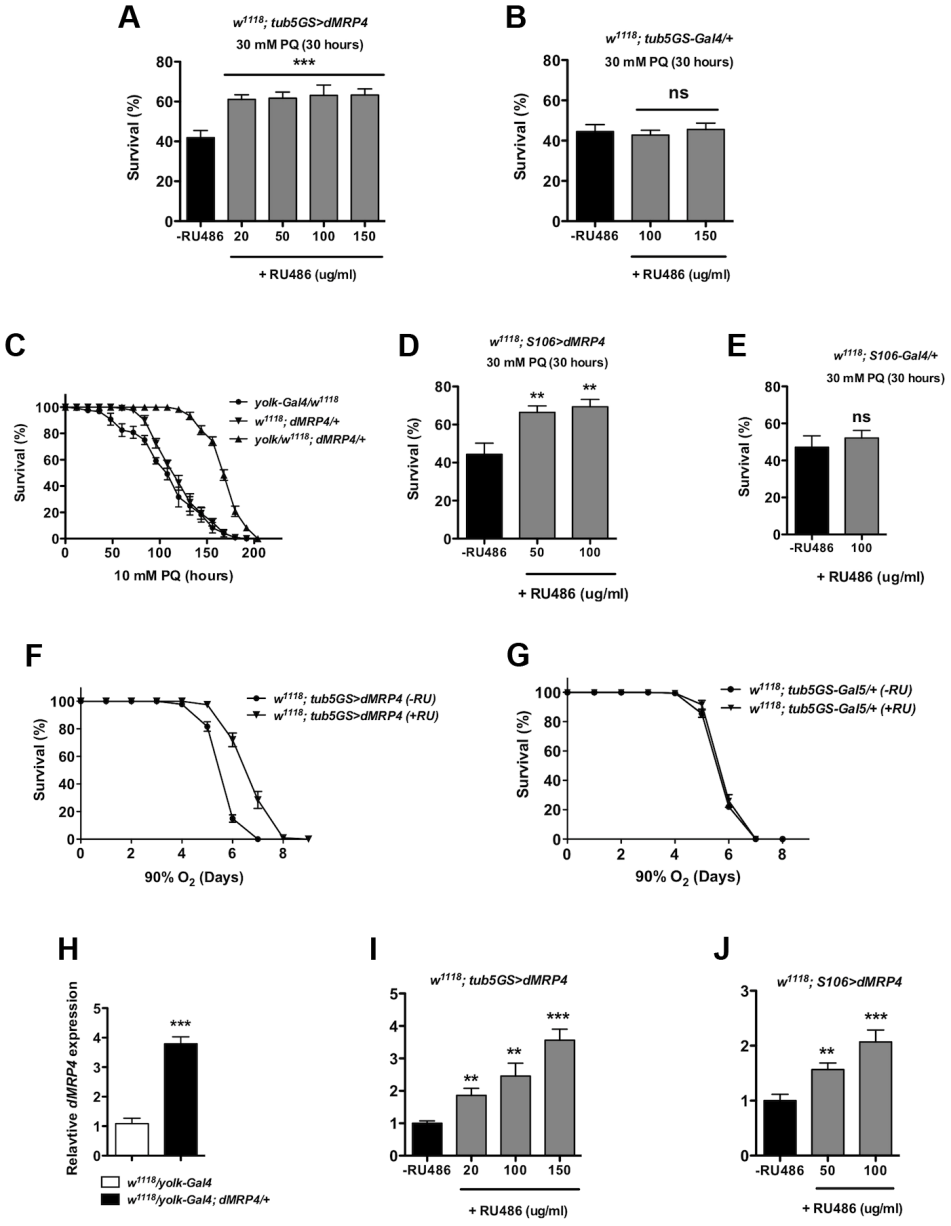
Elevated *dMRP4* expression increases oxidative resistance. Overexpression of *dMRP4* globally (A and F) or tissue-specifically (C–D), significantly promoted adult fly survival of paraquat (PQ)-induced oxidative stress. Since *yolk-Gal4* is expressed specifically in the female fat body, female flies were used in the *yolk>dMRP4* experiment (C). Male flies were otherwise used in all other experiments. (H–J) qt-PCR analysis of *dMRP4* induction by different Gal4 drivers. Concentrations of paraquat and RU486 used in individual experiment were indicated, except for (F–G) where concentration of RU486 used was 150 ug/ml. Student's *t*-test was used in (E and F) and ANOVA was used in (D) and (I–J). * *p*<0.05, ** *p*<0.01, *** *p*<0.001. ns: No significance (*p*>0.05). Sample size: (A) *tub5GS>dMRP4* [75], n = 160; *tub5GS>dMRP4* (+RU), n = 180; (B) *tub5GS-Gal4/+* [75], n = 160; *tub5GS-Gal4/+* (+RU), n = 160; (C) *yolk-Gal4/w^1118^*, n = 160; *dMRP4/+*, n = 160; *yolk-Gal4/w^1118^; dMRP4/+*, n = 160; (D) *S106>dMRP4* [75], n = 180; *S106>dMRP4* (+RU), n = 180; (E) *S106-Gal4/+* [75], n = 160; *S106-Gal4/+* (+RU), n = 160. (F) *tub5GS>dMRP4* [75], n = 200; *tub5GS>dMRP4* (+RU), n = 200; (G) *tub5GS-Gal4/+* [75], n = 200; *tub5GS-Gal4/+* (+RU), n = 180.

If *dMRP4* is essential for oxidative resistance in *Drosophila*, an increased *dMRP4* expression may increase oxidative resistance in wild-type flies. To test this hypothesis, we used the RU486-system to test the role of *dMRP4* overexpressing in paraquat resistance. Adult flies carrying *tub5GS>dMRP4*, after being fed with RU486 for *dMRP4* induction (Fig. 4I), significantly improved survival rates following acute treatment with paraquat (30 mM) compared to control flies (Fig. 4A). Importantly, RU486 feeding itself had no effect on survival under the same condition (Fig. 4B). These experiments underline the protective role of *dMRP4* from paraquat challenge. It also implies that this protection does not need *dMRP4* to be elevated before reaching adulthood.

Because mammalian MRP4 has been implicated in protecting the liver from oxidative stress [25], [26], we sought to investigate whether it was also the case in *Drosophila*. *Drosophila* fat body is an analogous tissue to mammalian liver and white adipose tissue [41], [42]. *yolk-Gal4* is expressed specifically in the female fat body [43]. We tested whether overexpression of *dMRP4* in the fat body could provide overall protection against oxidative damage to the whole fly. Induction of *dMRP4* in female fat body by *yolk-Gal4* led 4-fold increase in the *dMRP4* transcript (Fig. 4H) and rendered flies much more tolerant to paraquat treatment as compared to controls (*yolk-Gal4/+* or *dMRP4/+*) (Fig. 4C, Log-rank test, *p*<0.01). Similarly, overexpression of *dMRP4* by *S106-Gal4*, an inducible driver expressed predominantly in adult fat body [40], [44], [45], significantly increased survival of paraquat-fed flies in the presence of RU486 (Fig. 4D). Again, RU486 treatment showed dose-dependent induction of *dMRP4* expression (Fig. 4J) but played no role in mortality under the same condition (Fig. 4E). Thus, the *Drosophila* fat body appears to be an important tissue for *dMRP4* to sustain paraquat-induced oxidative stress. Furthermore, the protective role of *dMRP4* under paraquat challenge is applicable for both sexes.

The anti-oxidative effect of *dMRP4* on lifespan was further tested by exposing flies to hyperoxia. Flies overexpressing *dMRP4* by RU484 induction clearly lived longer under 90% oxygen environment compared to controls (Fig. 4F, Log-rank test, *p*<0.001). We conclude that wild-type *dMRP*4 function is to promote resistance to oxidative stress in *Drosophila*.

### *dMRP4* regulates normal lifespan

**Figure 5:**
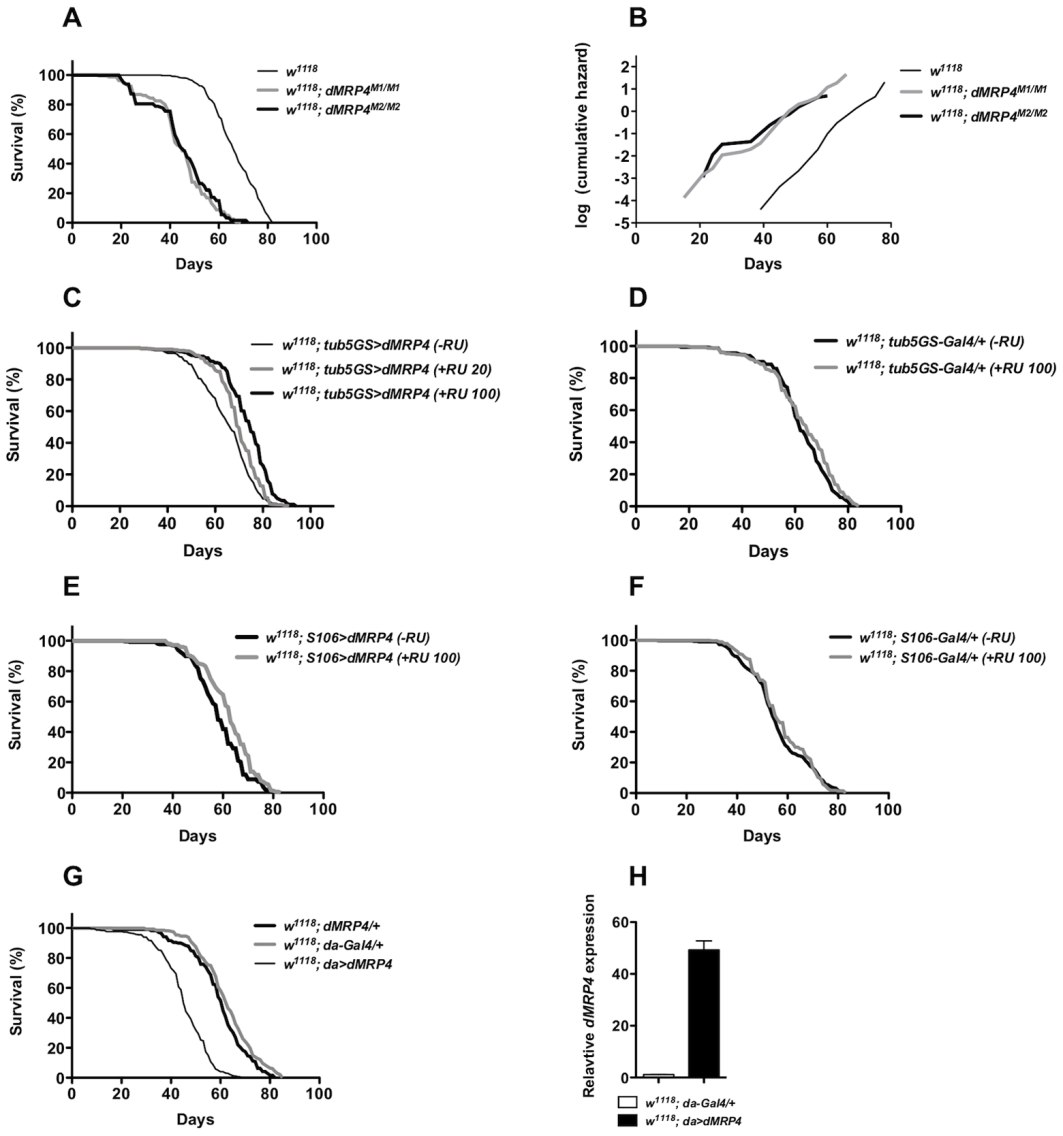
*dMRP4* affects lifespan. (A) Lifespan of adult flies lacking *dMRP4*. Survival was presented as mean of at least 300 males with different genotypes. Homozygous *dMRP4* mutant flies lived significantly shorter than their sibling controls (Log-rank test, *p*<0.0001): the mean lifespan (50% mortality) was 45 days for *dMRP4^M2/M2^* and 45 days for *dMRP4^M2/M1^*, respectively, compared to 66 days for wild-type control (*w^1118^*); the maximum lifespan (90% mortality) was 63 for *dMRP4^M2/M2^* and 60 days for *dMRP4^M2/M1^*, respectively, compared to 78 days for wild-type control. (B) Analysis of age-specific mortality. The log cumulative hazard plots were presented for different genotypes of survival data. The ratio of slopes from Partial Slopes Rank-Sum Test: *w^1118^* vs *dMRP4^M1/M1^* = 1.24 (*p* = 0.2005), *w^1118^* vs *dMRP4^M2/M2^* = 1.32 (*p* = 0.0947), *dMRP4^M1/M1^* vs *dMRP4^M2/M2^* = 1.07 (*p* = 0.7182). (C) Lifespan of adult flies with genotype *tub5GS>dMRP4* (*tubulin5-GS-Gal4/EP3177*) between treatments: −RU486 group, mean = 64 days, maximum = 78 days (n = 383), +RU486 group (100 ug/ml), mean = 74 day, maximum = 84 (n = 427), and +RU486 group (20 ug/ml), mean = 70 days, maximum = 82 days (n = 438). The cohort was derived from a combination of two independent cohorts (about 200 flies for each cohort) which were conducted over different time periods, and the individual cohorts were similar to each other. (D) Effects of RU486 treatment on control groups. *tub5GS-Gal4/+* (−RU486), mean = 62 days (n = 300) and *tub5GS-Gal4/+* (+RU486 100 ug/ml), mean = 62 days (n = 300). (E) Lifespan of adult flies with genotype *S106>dMRP4* (*S106-Gal4/+; EP3177/+*) between treatments: −RU486, mean = 60 days (n = 408), or +RU486, mean = 62 days (n = 460). (F) Effects of RU486 treatment on control groups. *S106-Gal4/+* (−RU486), mean = 54 days (n = 300) and *S106-Gal4/+* (+RU486 100 ug/ml), mean = 55 days (n = 300). (G) Lifespan of adult flies with genotype *da>dMRP4* was compared to the parent controls. The mean lifespan of these flies was 62 days for *da-Gal4/+* (n = 340), 61 days for *dMRP4/+* (n = 320), and 45 days for *da>dMRP4* (n = 380). The maximum lifespan was 77 days for *da-Gal4/+*, 77 days for *dMRP4/+*, and 56 days for *da>dMRP4*. (H) qt-PCR analysis of the *dMRP4* expression driven by *da-Gal4*.

Aging shares many features with oxidative stress [1]. The free radical theory has proposed a link between aging and oxidative stress [46], [47]. Recent studies from genetic manipulation of many genes in *Drosophila* have presented evidence that resistance to oxidative stress genetic often correlate with increased lifespan [6], [48], [49], [50]. Since manipulation of *dMRP4* can influence lifespan under oxidative stress, it would be important to examine whether *dMRP4* regulates lifespan under non-stress conditions. We observed that mutations in *dMRP4* dramatically caused a shortened normal adult lifespan (Fig. 5A, Log-rank test, *p*<0.0001). In particular, *dMRP4^M2/M2^* flies had a mean lifespan (as measured by 50% survival) of 45 days and a maximum lifespan (as measured by the 90 percent survival) of 60 days. Compared to wild-type controls, *dMRP4^M2/M2^* flies had a major reduction in the mean lifespan of about 47% and a decrease in maximum lifespan of 24% (Fig. 5A). Similar results were observed with *dMRP4^M1/M1^* flies (Fig. 5A). The overall mortality rates of these groups were compared using Partial Slopes Rank-Sum Test [51] over the linear portion of the increase in mortality. Despite an apparent initiation of early mortality before day 30 in survival of *dMRP4* mutants, there was no significant difference in slopes between the mutants and wild type (Fig. 5B), indicating that loss of *dMRP4* decreased lifespan by lowing the whole mortality trajectory, but not the rate of increase in mortality with age. Thus, although *dMRP4* is not required for normal development, it is required for normal lifespan under non-stress conditions.

Since flies overexpressing *dMRP4* were more resistant to oxidative stress, we tested whether overexpressing *dMRP4* would be sufficient to extend lifespan. RU486-mediated overexpression was used to minimize the influence of genetic background on lifespan assays. RU486-fed *tub5GS>dMRP4* flies lived significantly longer than their siblings without RU486 feeding (Fig. 5C, Log-rank test, *p*<0.0001). The lifespan extension by *tub5GS>dMRP4* expression appeared to be correlated with the dose of RU 486. In one case, the mean lifespan was extended to 16% and the maximum lifespan to 8% (Fig. 5C, RU486 100 ug/ml). In the other case, when flies were fed with 20 ug/ml RU486, this group of flies showed only about 9% of increase in the mean lifespan and 5% of increase in the maximum lifespan, even though their overall lifespan appeared to significantly increase (Fig. 5C, Log-rank test, *p*<0.0001). Increased lifespan was not due to chronic RU486 treatment because no significant difference in lifespan was seen between treated or untreated *tub5GS-Gal4* groups (Fig. 5D, Log-rank test, *p* = 0.3). We conclude that another *dMRP4* function is to promote normal lifespan in *Drosophila*.

In these experiments the lifespan extension clearly correlated with increased expression of *dMRP4*, but it remained unclear whether tissue-specific *dMRP4* overexpression was sufficient to extend lifespan and whether the overall levels and/or timing of such expression would be critical. Interestingly, *S106>dMRP4* flies treated with RU486 did not live longer (Fig. 5E, Log-rank test, *p* = 0.37) even though the fat body-specific expression of *dMRP4* did show resistance to paraquat, suggesting that there might be different requirements between resistance to oxidative stress and lifespan extension. Again, RU486 treatment showed no difference between parallel controls (Fig. 5F, Log-rank test, *p* = 0.09). Moreover, high levels of ubiquitous d*MRP4* expression by *da-Gal4* throughout development were not beneficial and instead, there was a negative correlation with lifespan (Fig. 5G, Log-rank test, *p*<0.0001). These observations suggest that in order for *dMRP4* overexpression to be beneficial for lifespan extension, the spatial and temporal such expression with proper levels have to be tightly controlled.

### *dMRP4* regulates the basal transcription of stress- and longevity- associated genes

**Figure 6:**
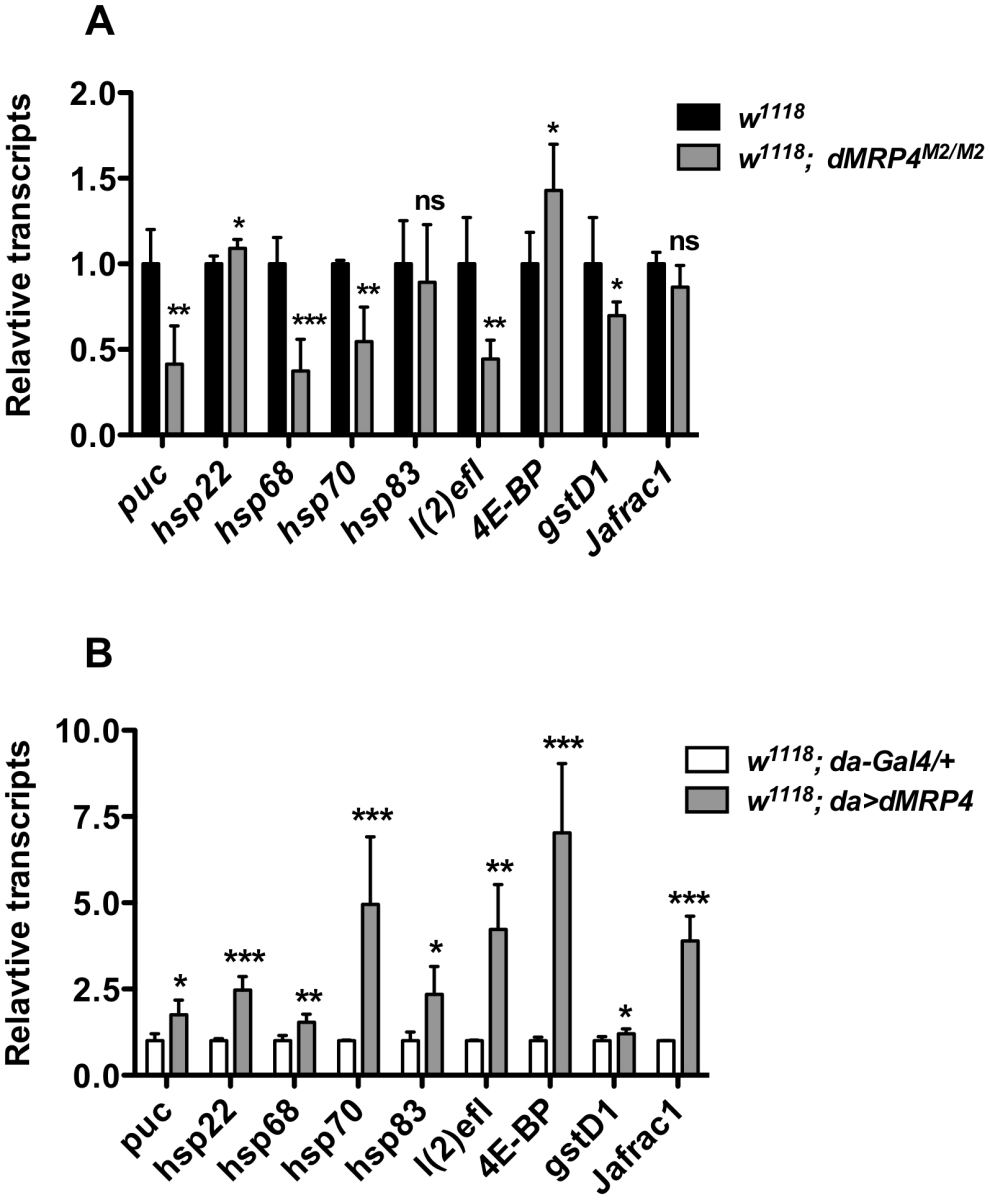
*dMRP4* regulates expression of some stress- and aging-related genes. (A) *dMRP4* was required for basal expression of several stress- and aging-related genes. The mRNA levels of these genes were compared between *w^1118^* and *dMRP4* mutant (*dMRP4^M2/M2^*) by qt-PCR analysis. (B) Elevated expression of *dMRP4* increased the basal expression of stress- and aging-related genes. The mRNA levels of these genes were compared between control Gal4 (*da/+*) and *dMRP4* overexpression (*da>dMRP4*) by qt-PCR analysis. Data was presented as means ± SD from 3–5 independent experiments. Student's *t*-test: * *p*<0.05, ** *p*<0.01, *** *p*<0.001. ns: No significance (*p*>0.05).

In order to learn the molecular mechanism by which *dMRP4* regulates lifespan, we selectively studied transcription profiling of several genes whose expression changes have been linked to both aging and stress [1]. Among five *hsp* (heat shock protein) genes examined, expression of three genes, *hsp68*, *hsp70* and *l(2)efl* (*lethal (2) essential for life*, a small *hsp* gene) was severely down-regulated in *dMRP4* mutant flies (Fig. 6A), while they were significantly up-regulated when *dMRP4* was overexpressed (Fig. 6B). Overexpression of *dMRP4* was also sufficient to increase expression of other two *hsp* genes, *hsp22* and *hsp83* (Fig. 6B). Since *l(2)efl* is a known target of *dFOXO* (*Drosophila* forkhead transcription factor) in lifespan regulation [52], it raised the possibility that *dMRP4* might regulate expression of other *dFOXO*-dependent genes. Indeed, expression of the *dFOXO* target gene *thor*, which encodes 4E-BP (eIF4E binding protein), was also greatly enhanced when *dMRP4* was overexpressed. Since both *thor* and *hsp68* are target genes of JNK signaling [6], [52], we further examined expression patterns of several other JNK targets (Fig. 3A–D). Like *hsp68*, basal expression of *puc* and *gstD1* was down-regulated in *dMRP4* mutant flies and was up-regulated with *dMRP4* overexpression (Fig. 6A–B). Furthermore, basal expression of *Jafrac1* was increased when *dMRP4* was overexpressed, even though its expression was not affected by *dMRP4* mutation under normal condition. Thus, in addition to regulating the JNK-dependent gene expression under oxidative stress, *dMRP4* also regulates the basal transcription of such genes under normal conditions.

Increased expression of *hsp22* [53], *hsp68* [6], [54], *hsp70* [55], *l(2)efl* [52], *Jafrac1* [54], [56], has been reported to increase *Drosophila* lifespan. We hence suggest that increased expression of these genes by elevated *dMRP4* expression may account for, at least in part, the *dMRP4*-mediated lifespan extension.

### *dMRP4* regulates the aging process

**Figure 7:**
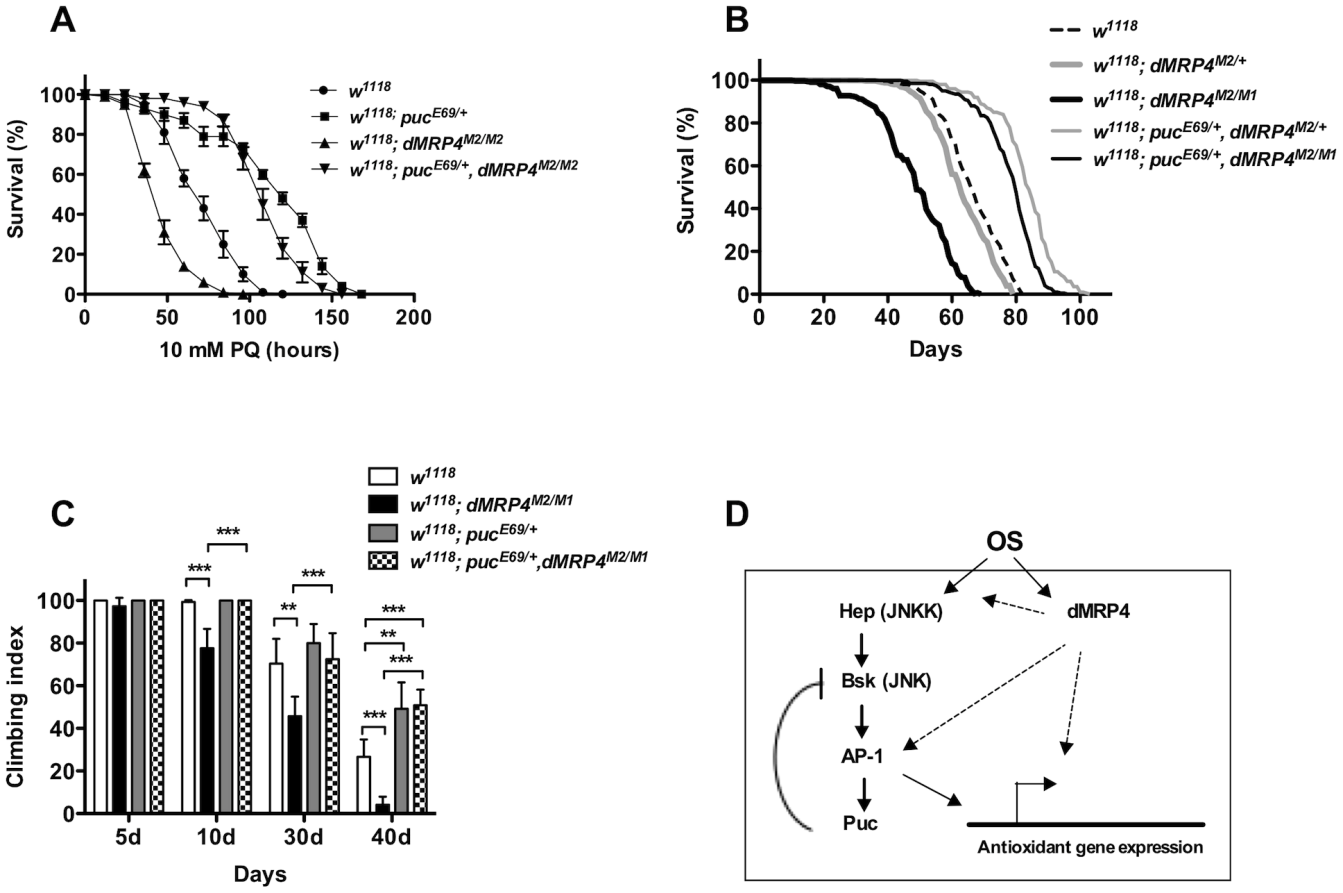
Extending lifespan of *dMRP4* deficiency by a mild increase in JNK signaling under both paraquat resistance and normal condition. Flies heterozygous for *puc* (*puc^E69/+^*) were significantly resistant to paraquat-induced oxidative stress (A) and had a remarkably longer lifespan under non-stress condition (B) compared to controls. (A) The lifespan of *dMRP4* mutant flies (*dMRP4^M2/M1^*) under paraquat stress was compared to control (*w^1118^*) and *puc*, *dMRP4* double mutant flies (*puc^E69/+^*, *dMRP4^M2/M1^*). Each group represented 180 flies. (B) The lifespan of *dMRP4* mutant flies (*dMRP4^M2/M1^*) under normal condition was compared to controls (*dMRP4^M2/+^* and *w^1118^*) and *puc*, *dMRP4* double mutant flies (*puc^E69/+^*, *dMRP4^M2/M1^*). The mean lifespan (50% mortality) was 48 days for *dMRP4^M2/M1^* (n = 360), 62 days for heterozygous control *dMRP4^M2/+^* (n = 360), and 67 days for *w^1118^* (n = 300), 83 days for *puc^E69/+^*, *dMRP4^M2/+^* (n = 320), and 79 days for *puc^E69/+^*, *dMRP4^M2/M1^* (n = 360). The maximum lifespan (90% mortality) was 62 days for *dMRP4^M2/M1^*, 75 days for heterozygous control (*dMRP4^M2/+^*), 93 days for flies *puc^E69/+^*, *dMRP4^M2/+^*), 77 days for *w^1118^*, and 88 days for flies (*puc^E69/+^*, *dMRP4^M2/M1^*). (C) The locomotor defect, indicated by negative geotaxis performance, of *dMRP4* mutant flies was completely restored by *puc^E69/+^*. Each column was derived from a pool of 80–100 male flies with indicated ages. Student's *t*-test: ** *p*<0.01, *** *p*<0.001. Error bars were S.D. (D) A model for role of *dMRP4* in JNK-mediated oxidative resistance and lifespan extension. Paraquat-induced oxidative stress (OS) was sensed through JNK and dMRP4, respectively. The OS signaling may then be converged at levels of the AP-1 transcription factors, the major target of JNK, because *dMRP4* is necessary and sufficient for transcription of some JNK-dependent antioxidant genes and because genetic manipulation of *puc* can fully rescue *dMRP4* mutant phenotypes under both oxidative and normal conditions. However, it is also possible that *dMRP4* is required for JNK to fully respond to OS at any levels upstream of AP-1. Involvement of *dMRP4* in regulation of other JNK-independent antioxidant genes cannot be excluded. The solid arrows represent the work from published studies, and dash arrows indicate the work from this study.

Increasing age is accompanied with physiological decline. The locomotor decline is one of prominent physiological changes as they grow older. The climbing ability, measured by negative geotaxis, of adult fly reflects a function of age in *Drosophila* [57], [58]. To determine whether the onset of aging associated with *dMRP4*, we performed a negative geotaxis test for flies with different ages. Although there was no difference in negative geotaxis behavior between 5-days old *dMRP4* mutant and wild-type adults, the age-associated functional decline became visible in *dMRP4* mutant flies already at day 10 of adulthood, at a time when no mortality was seen regardless of mutant or wild-type controls (Fig. 7C). By age 40 days, although there was a progressive functional decline in the control group, it was clearly worse in *dMRP4* mutant groups (*w^1118^* vs *dMRP4^M2/M1^*, Fig. 7C). Thus, the functional decline as they aged was faster in *dMRP4* mutants than in controls.

### Activation of JNK signaling rescues *dMRP4* deficiency

Activation of JNK signaling can increase stress resistance and extend lifespan in both *Drosophila* [6], [52], [59], and *C.elegans* [60]. Our observations (Fig. 3A–D, Fig. 6A–B) suggest that the deficiency in basal transcription and stress response of JNK signaling may be an important cause for loss of stress tolerance and normal lifespan with *dMRP4* mutant flies. If this were the case, increasing JNK signaling might be expected to correct *dMRP4* deficiency. We tested this hypothesis by recombination of a *puc^E69^* chromosome into the d*MRP4* mutant background. *puc^E69^/+* flies were more resistant to paraquat and lived longer under normal conditions [6] (Fig. 6A and B). When *dMRP4* mutant flies also heterozygous for *puc^E69^* were challenged with paraquat, they behaved like *puc^E69^/+* flies alone: they lived significantly longer not only than *dMRP4* mutant flies, but also longer than wild-type controls (Fig. 7A, *p*<0.01). Consistent with a previous report [6], *puc^E69^/+* flies extended normal lifespan (27% mean lifespan and 24% maximum lifespan) of control flies (*dMRP4^M2/+^*) under non-stress conditions (Fig. 7B, *p*<0.0001). More strikingly, the *puc*, *dMRP4* double mutant flies remarkably extended the mutant mean lifespan by 61% (*dMRP4^M2/M1^* vs *puc^E69/+^*, *dMRP4^M2/M1^*) and maximum lifespan by 42% (Fig. 7B, *p*<0.0001). These results demonstrate that *dMRP4* deficiency in stress resistance and lifespan regulation is correlated with a defect in JNK signaling. These results also place *puc* genetically in epistatic interaction with *dMRP4* in both stress resistance and lifespan regulation.

We tested whether the functional decline with age might also be associated with JNK activity by comparing the climbing ability between wild-type and *puc^E69^/+* flies. Increased JNK signaling did not appear to benefit wild-type flies before 30 days of age, as climbing tests did not reveal a significant difference in locomotor function between wild-type and *puc^E69^/+* flies (Fig. 7C). However, after 40 days of age, increased JNK activity indeed improved climbing ability, and therefore functional aging in wild-type flies (*w^1118^* vs *puc^E69^/+* in the 40 d group, Fig. 7C), suggesting that JNK activity is required for fitness of older flies. We then tested whether the age-associated functional decline of *dMRP4* mutants could be caused by impaired JNK signaling as well. The climbing ability of *puc*, *dMRP4* double mutant flies was restored to the level comparable to that of wild-type flies in the first 30 days of age. Therefore, early functional decline of *dMRP4* mutants is possibly associated with a decline of JNK signaling (Fig. 7C). Furthermore, by age of 40 days, *puc*, *dMRP4* double mutant flies behaved like *puc^E69^/+* flies, showing better climbing performance even over wild-type flies (Fig. 7C). Thus, the JNK activity can seemingly rescue all defects that are associated with *dMRP4* phenotypes. We conclude that *dMRP4* plays a critical role in regulation of JNK-mediated oxidative resistance and aging process.

## Discussion

The MRP4 subfamily and its homologs have not been reported in any lifespan-related studies including genome-wide surveys. In this study, we have investigated the physiological function of *dMRP4* gene in *Drosophila*. A main finding from our work is that *dMRP4* regulates lifespan under both normal conditions and oxidative stress, concomitantly with changes of JNK activity *in vivo*. Our main finding is based on several observations: *First*, *dMRP4* is required for induction of some JNK-dependent genes in response to paraquat-induced oxidative stress. *Second*, elevated *dMRP4* expression stimulates basal transcription of some JNK-dependent genes downstream of JNK signaling. *Third*, increased JNK activity in *dMRP4* mutant background can rescue *dMRP4*-related phenotypes identified in this work, supporting our hypothesis that *dMRP4* may regulate oxidative resistance and lifespan, at least in part, through JNK signaling.

The finding that *dMRP4* has a role in lifespan is particularly intriguing because we are able to show for the first time that a drug transporter like MRP4 is involved in lifespan regulation. Like *Drosophila dMRP4*, *MRP4* KO mice show no visible phenotype [11], [12], [13], [14], and *mrp-4* knockdown in *C.elegans* with RNAi results in no observed phenotype either [61], [62]. These observations together suggest that MRP4 and its homologs across species do not contribute to normal development in the animal world. However, unlike in other species, we found that the *Drosophila dMRP4* is required for adult lifespan. Flies deficient for *dMRP4* live significantly shorter, under both stressful and normal conditions. Subsequently, our work reveals that *dMRP4* acts as a modulator of a network of gene expression since loss- or gain-of *dMRP4* function leads to major changes in the transcriptional profiling of a number of genes that may contribute to lifespan regulation. Therefore we suggest that gene expression changes mediated by *dMRP4* may represent a molecular mechanism by which *dMRP4* regulates lifespan. For instance, *hsp* genes have been implicated in regulation of both stress resistance and lifespan extension [4], [63], and are among the best-known biomarkers of aging in *C.elegans* [63], [64], in *Drosophila* [1], [65], and perhaps even in humans [66]. Given the fact that the expression of *hsp* reporters in young individual flies has been observed to be partially predictive of remaining lifespan [65], down-regulation of several *hsp* gene expression (i.e. *hsp68*, *hsp70*, *l(2)efl*) in *dMRP4* mutant background could explain the shorter lifespan of these flies, while their up-regulation (i.e. *hsp22*, *hsp68*, *hsp70*, *hsp83*, *l(2)efl*) at a young age by *dMRP4* overexpression may help protect against oxidative stress and extend lifespan of wild-type flies. This scenario is consistent with previous notions that genes are involved in stress responses generally share similar involvement with aging [1].

In addition to *hsp* genes, the interaction of *dMRP4* with JNK signaling may provide an alternative mechanism to explain *dMRP4* functions. Because the JNK pathway is known to be crucial in stress resistance and aging, impairment of JNK signaling in *dMRP4* mutant flies, indicated by transcriptional down-regulation of several known JNK-related effecters, could result in *dMRP4*-associated phenotypes. The acute phenotype is seen particularly when the animal faces stressors such as paraquat-induced oxidative stress, which recapitulates the phenotype shown by mutations in the JNK pathway [6]. The effect of the JNK pathway on lifespan has also been observed during aging under normal conditions. Flies with reduced JNK activity have a shorter lifespan [6], a phenotype similar to that seen in *dMRP4* mutant flies. Furthermore, some downstream effectors in the JNK pathway also exhibit phenotypes that are reminiscent of *dMRP4*. For instance, loss of *Jafrac1* function leads to an exaggerated sensitivity to paraquat-induced oxidative stress and a shortened lifespan, while overexpression of *Jafrac1* increases oxidative resistance and extends lifespan [56]. Interestingly, expression of *Jafrac1* transcription is down-regulated in the *dMRP4* mutant in response to oxidative stress (Fig. 2D) and is up-regulated by *dMRP4* overexpression (Fig. 6B). How *dMRP4* regulates *Jafrac1* remains to be investigated. One possible scenario is that *dMRP4* executes its functions through interacting with JNK signaling to modulate the expression of downstream effectors such as *Jafrac1* especially that the expression of *Jafrac1* itself is regulated by JNK signaling [56]. After all, the most compelling evidence for the relationship between *dMRP4* and JNK signaling comes from our genetic epistatic assays. When JNK signaling is enhanced in *dMRP4* mutant background, all *dMRP4*-related defects are restored, and *puc*, *dMRP4* double mutant flies now phenocopy *puc^E69/+^* flies, clearly proving that JNK signaling plays a central role in realizing *dMRP4* functions. Our work also suggests that promoting lifespan by increasing JNK signaling may be a result of its ability to antagonize oxidation on macromolecules, thereby postponing aging. Compared to JNK signaling, the effect of increased *dMRP4* expression on lifespan extension seems less dramatic. Yet this phenotype, together with the results showing that loss- or gain-of JNK function does not alter *dMRP4* expression, indicates that *dMRP4* functions as a modulator of, but not a component within, JNK signaling. Furthermore, if *dMRP4* is one of upstream modulators of JNK/Puc signaling, it is conceivable that its overexpression cannot entirely recapitulate the effect of JNK/Puc activation and consequently, it may not be as effective as a direct manipulation of JNK/Puc signaling with respect to lifespan.

Together our results, we propose a working model to summarize how *dMRP4* executes its functions in conjunction with JNK signaling (Fig. 7D). Future work needs to explore how a transmembrane protein such as dMRP4 could integrate its signal into the JNK pathway under both stress and normal conditions.

Although in human and mammalian models of cholestasis, MRP4 has been implicated in providing protection against oxidative stress, the genetic basis for this resistance has not yet been addressed. Therefore, the connection between tissue oxidative stress, survival of the animal, and the physiological function of MRP4, has been lacking. In this work we show that overexpression of *dMRP4* in *Drosophila* fat body, the equivalent tissue of mammalian liver and white adipose tissue, can confer oxidative resistance to the whole animal, suggesting a functional importance of *dMRP4* in the fat body in the protection of *Drosophila* against oxidative stress. *Drosophila* fat body has recently been reported as a primary site of lipid oxidative damage after paraquat treatment [67]. *dFOXO*, whose expression is predominately restricted to the fat body, appears to regulate sensitivity of paraquat-induced oxidative damage and age-associated degeneration of behavioral rhythms through this tissue [68]. Furthermore, overexpression of *dFOXO* in the adult fat body can increase stress resistance and retard aging process [44], [69], supporting the physiological role of fat body in stress defense for the whole organism. Strikingly, we show in this work that expression of two targets of *dFOXO*, *l()efl* and *thor*, are greatly induced when *dMRP4* is overexpressed, raising the intriguing possibility that *dMRP4* may promote stress resistance and lifespan extension by activation of *dFOXO*, for instance through JNK signaling [52]. However, unlike the finding that global induction of *dMRP4* can promote lifespan, we have not observed a significant lifespan extension when *dMRP4* overexpression is restricted in fat body. This observation suggests that the ability of stress resistance may not be an absolute factor associated with longevity in a particular tissue. It is also possible that in order for *dMRP4* to benefit for longer life, more tissues with its elevated expression need to be involved. Our studies in fact have not ruled out the roles of *dMRP4* in tissues other than the fat body to survival even under oxidative stress.

The main function of MRP4 family is known for their ability to transport a variety of diverse endogenous and xenobiotic compounds. An interesting speculation could be raised as to whether *dMRP4* might function simply as a transport in paraquat resistance. In this scenario, flies deficient in *dMRP4* might not be able to efficiently exclude paraquat out of cells, thereby leading to substrate-related toxic effects. However, this assumption would hardly explain why flies deficient in *dMRP4* lose their resistance to hydrogen peroxide and hyperoxia. In addition, there is no report for paraquat as a potential substrate of any MRP4 members thus far.

The deteriorate influence by *da>dMRP4* overexpression is notable because this phenotype has not been seen in overexpression studies of mammalian MRP4. Although use of the whole animal in this study clearly differs from use of cultured cells in mammalian researches, it is more likely that high levels of *dMRP4* expression may interfere with normal development, resulting in a pleotropic impact on later assays. An early report did observed that overexpression of two EP lines, which all targeted *dMRP4*, in larvae caused neuromuscular phenotypes [70].

Given the considerable conservation of pathways between *Drosophila* and mammals, it will be interesting to test if manipulating *MRP4* in mammalian liver cells could confer resistance to the liver, or even to the whole animal subjected to chemotherapy-induced oxidative stress. Finally, our proposed mechanism that interactions between *dMRP4* and JNK signaling may shed new light on the clinic problems for long-lived cancer cells with drug resistance due to elevated expression of MRP including MRP4 proteins.

## Materials and Methods

### Fly strains and genetics

*EP3177* and *EP3655* were described previously [31]. Other stocks: *w^1118^*, *w; TM3,Sb,Ser/TM6B,Tb*, *w; Sco/CyO; MKRS/TM6B,Tb*, *daughterless* (*da*)*-Gal4*, *S106-Gal4*, *puc^E69^*, *UAS-Bsk^DN^ and UAS-Hep^Act^* strains were obtained from Bloomington stock center. These strains have been backcrossed to *w^1118^* for 8–10 times before experiments. *yolk-Gal4* [43] was kindly provided by Norbert Perrimon and was backcrossed into *w^1118^* background for 8 times. *Actin-GeneSwitch-Gal4* (*actGS-Gal4*, [71], [72]) was a gift from Dirk Bohmann. *tublin5-GeneSwitch-Gal4* (*tub5GS-Gal4*, [73]) was a gift from Scott Pletcher. These Gal4 strains have been backcrossed into *w^1118^* background for 6 times before use. Flies were raised on standard *Drosophila* food (per liter: 17.3 g of yeast, 73.1 g of cornmeal, 10 g of soy flour, 77 ml of light corn syrup, 4.8 ml of propionic acid, and 5.7 g of agar).

To generate *dMRP4* mutant flies, two independent EP lines, *EP3177* and *EP3655* were first backcrossed into *w^1118^* background for 8 times. EP males were crossing to *w^1118^*; *Δ2-3 Sb/TM3* females that provides with transposase. Males with mosaic color eyes were excised and subsequently balanced with *w^1118^; TM3,Sb,Ser/TM6B,Tb* strain. The balanced excisions were then repeatedly backcrossed via the balancer strain for 8 times to establish excision stocks. They were identified by loss of the expression of the mini-white gene. The genomic deletions were determined by sequencing with specific primers spanning the EP insertion region. Two deletions obtained had truncated the 5′-end of putative *dMRP4* transcript, which was designated as *dMRP4* mutation 1 (*w^1118^; dMRP4^M1^*) and *dMRP4* mutation 2 (*w^1118^*; *dMRP4^M2^*) (Fig. 2A). *dMRP4^M1^* was excised from *EP3655*, which inserted at 47 bp from the transcription start site of the predicted gene *CG14709*, resulting a 2.7 kb deletion that removed 1179 bp upstream of *dMRP4* transcript and a 1521 bp region including 585 bp of the entire exon 1 encoding the first 25 amino acids of the protein, as well as 936 bp of the intron 1. *dMRP4^M2^* was resulted from an excision of *EP3177*, which inserted at 88 bp from the transcription start site of the predicted gene *CG14709*. This led to a 3 kb deletion that has removed 2117 bp upstream of *dMRP4* transcript and an 883 bp region spanning the entire exon 1 and part of intron 1.

The *puc^E69^*, *dMRP^M2^* recombination strain was generated by recombination of *puc^E69^* and *dMRP^M2^* onto the same 3^rd^ chromosome. Both the balanced *puc^E69^* and *dMRP^M2^* were repeatedly backcrossed via *w^1118^; TM3,Sb,Ser/TM6B,Tb* for 8 times before the recombination experiments. The presence of both mutations after meiotic recombination was verified by genetic cross and by PCR with specific primers. Resultant *puc^E69/+^*, *dMRP^M2/M2^* double mutants were then continuously backcrossed via *w^1118^; TM3,Sb,Ser/TM6B,Tb* for more than 10 times and were kept with the balancer as a parent stock.

To induce *dMRP4* overexpression, adult flies carrying different Gal4 drives were crossed to homozygous *EP3177* lines. For RU486 induction, a 25 mg/ml RU486 (mifepristone, Sigma) stock solution made in 100% ethanol was diluted with water for desired concentrations. 250 ul of diluted RU486 solution was added onto the surface of standard fly food. This “on food” method has been shown to be simple and effective over other RU486 supply methods [74]. The vials were allowed to dry for 24 hours before use. The same solution without RU486 was added to fly food for control experiments.

### Paraquat treatment

In most experiments, three to four day-old males, grouped with 20 flies per vial, were fed on a 3 mm Whatmann paper soaked with 10 mM paraquat (*N*,*N*′-dimethyl-4,4′-bipyridinium dichloride, Sigma) in 5% sucrose/PBS. Flies of different genotypes were also fed only with 5% sucrose/PBS as experimental controls. Under this condition all flies can live up for 10 days perfectly. Scores were done every 12 hours for the number of dead flies. Fresh paraquat was added daily. All tests were performed at 25°C. Flies were not starved before adding paraquat in this test to avoid unnecessary stress. Survival comparisons were analyzed by Kaplan–Meier Log-rank Test using Graph Pad Prism4. *p*<0.05 was considered statistically significant.

In RU486-induced experiments, 20 adult males (2–4 days old) per vial were fed with different concentrations of RU486 for 4–6 days. They were then transferred on a 3 mm Whatmann paper soaked with 30 mM paraquat in 5% sucrose for acute survival test, or with 10 mM paraquat in 5% sucrose for mRNA induction at 24 hours. Control flies were from the same collection and were treated in parallel.

For RNA, all samples were collected at the end of treatments and were immediately frozen in dry ice for RNA preparations.

### Hydrogen peroxide treatment

Eight day-old males were fed with different concentrations of hydrogen peroxide (v/v, Sigma) in 5% sucrose/PBS. Control flies were fed with 5% sucrose/PBS only. RNA for qt-PCR was extracted from these flies after 24 hours treatment. For survival tests, ten day-old males with different genotypes were fed with 3% hydrogen peroxide. Fresh hydrogen peroxide was added every day. Scoring and analysis were done essentially as described in paraquat treatment.

### Hyperoxia treatment

Eight day-old males, grouped with 20 flies per vial on regular food, were exposed to a steady flow of 95% or 90% oxygen bubbled through water in a sealed chamber. RNA for qt-PCR was extracted from these flies after indicated time points. For survival tests, twelve day-old males with different genotypes were treated with 90% oxygen as above. For RU486 induction, flies from the same breeding were divided into two groups, one group fed on food containing RU486 (150 ug/ml) and the other on normal food through the experiments. Flies were transferred to fresh vials every 2–3 days. Scoring was done every day.

### Lifespan

Flies were collected within 24 hours of eclosion and grouped into 20 males per vial. Tests were performed at 25°C. For each experiment, at least 200 flies of each genotype were tested.

For GeneSwitch experiments, males of genotypes *w^1118^*; *tub5GS-Gal4*, *w^1118^; actGS-Gal4*, *or w^1118^; S106-Gal4* were crossed to *w^1118^*; *EP3177* or *w^1118^* females, respectively. Male progeny from these crosses were aged for 3 days after eclosion, and then were divided into 20 flies per vial, with or without indicated concentrations of RU486 in food. Flies were transferred to fresh vials with or without RU486 every other day and dead flies were scored at the time of transfer.

All experiments were conducted at least two times from independent biological breeding. The maximum lifespan was the mean lifespan of last 10% of survival animals in each cohort.

### Negative geotaxis

10–20 male flies, ages from 5–40 days at 25°C, were transferred to a clean plastic vial, rested for 3 min, and then measured for bang-induced vertical climbing distance at room temperature (20–21°C). The performance was scored as percentage of flies crossing 7 cm within 10 seconds in a single vial, which was expressed as average of 5 repeated tests for a single vial. 80–100 flies were tested for each genotype at each time point.

### RT-PCR

Total RNA was isolated from whole flies using RNeasy Mini Kit (Qiagen, Maryland, USA) according to the manufacturer's instructions. cDNA synthesis was performed with oligo-dT and random primers using SuperScript III first-strand synthesis system (Invitrogen, Carlsbad, CA). Semiquantitative PCR was performed as described [31]. Real-time PCR was performed in duplicate using SYBR Green on an ABI 7900HT Real-Time PCR system (Applied Biosystems) according to the manufacture's protocol. All samples were analyzed from at least 3 independent of experiments. Data was normalized first to the level of the *rp49* mRNA prior to quantifying the relative levels of mRNA between controls and experimentally treated samples. All detailed primers are available upon request.

### Statistics

All survival data were analyzed by Kaplan–Meier Log-rank Test for overall survival and by the Student's *t*-test for mean and maximum lifespan using Graph Pad Prism4. The log mortality was determined by OASIS program [51]. Treated data were then plotted using Graph Pad Prism4. Other comparisons were determined either by Student's *t*-test or One way ANOVA followed by post hoc *t*-test. *p*<0.05 was considered statistically significant.
